# Effectiveness of acupuncture for cancer-related fatigue: a systematic reviews and meta-analysis

**DOI:** 10.3389/fonc.2026.1790238

**Published:** 2026-05-05

**Authors:** Hongxiao Zhang, Zhifeng Pan, Yuting Huang, Run Lin, Liying Wang, Tao Peng, Mingzhu Xu, Zining Guo, Shaoyang Cui

**Affiliations:** 1 Shenzhen Hospital (Fu Tian) of Guangzhou University of Chinese Medicine, Shenzhen, China; 2 The Sixth Clinical Medical School, Guangzhou University of Chinese Medicine, Guangzhou, China; 3 Department of Rehabilitation Medicine, Shenzhen Hospital, Southern Medical University, Shenzhen, China; 4 South China Research Center for Acupuncture and Moxibustion, Medical College of Acu-Moxi and Rehabilitation, Guangzhou University of Chinese Medicine, Guangzhou, China

**Keywords:** acupuncture, cancer-related fatigue, meta-analysis, quality of life, supportive care in oncology, systematic review

## Abstract

**Background/objectives:**

Acupuncture is recommended for the treatment of cancer-related fatigue (CRF), but the strength of evidence remains limited. This study aims to comprehensively update the evaluation of the effectiveness of acupuncture for CRF and clarify the latest strength of evidence.

**Methods:**

Eight databases were searched from inception to April 2025 to identify eligible randomized controlled trials (RCTs). The quality of the study was assessed using the Cochrane risk of bias tool version 2.0(ROB 2.0). Meta-analyses were performed using R studio 4.4 software and RevMan software (version 5.4). Subgroup analyses by acupuncture type were conducted to explore sources of heterogeneity and further elucidate the efficacy of different acupuncture modalities. Publication bias was assessed when applicable. Finally, evidence quality was rated using the GRADE system.

**Results:**

This meta-analysis included 28 RCTs. Risk of bias assessment indicated low overall study quality. The meta-analysis of the primary outcome(Piper Fatigue Scale, PFS) revealed that acupuncture intervention significantly improved fatigue levels compared to the control group (MD = -0.56, 95% CI: -0.74 to -0.38, I2 = 45%, P < 0.01). Subgroup analyses showed that different acupuncture type had distinct advantages, suggesting distinct targeted advantages. Other factors showed significant differences. Sensitivity analyses for multiple outcome measures yielded unstable results, with indications of publication bias. According to GRADE criteria, the most outcomes were rated as low or very low quality.

**Conclusions:**

Current evidence suggests that acupuncture may alleviate CRF. However, conclusive evidence supporting its therapeutic efficacy remains limited. Given the methodological concerns and low certainty of the available evidence, further high-quality studies are needed to confirm these findings.

**Systematic review registration:**

https://www.crd.york.ac.uk/prospero/, identifier CRD42024603184.

## 1 Introduction

CRF is defined as distressing, persistent, subjective physical, emotional, or cognitive fatigue or exhaustion associated with cancer or its treatment that is disproportionate to recent activity (1). Unlike ordinary fatigue, CRF is not relieved by rest and significantly impairs the quality of life in cancer patients (2). Epidemiological data indicate that nearly 70% of cancer survivors report suffering from CRF, with symptoms persisting for up to 10 years in some populations (3–7, 71). Furthermore, CRF is closely associated with the survival prognosis of cancer patients. It has been identified as an independent risk factor contributing to disease progression, particularly in breast and prostate cancer (8–10). Currently, the pathological mechanisms of CRF have not been fully elucidated. The mainstream pathogenic hypotheses include dysfunction of the hypothalamic-pituitary-adrenal axis (HPA) (11, 12), excessive release of pro-inflammatory cytokines (e.g., IL-6, TNF-α) (13, 14), and so on. Additionally, the development of CRF is closely associated with psychosocial factors (e.g., depression, anxiety) and physiological factors (e.g., anaemia, malnutrition, pain), which significantly complicate the treatment of CRF (15). Currently, clinical treatments of CRF are divided into pharmacological and non-pharmacological approaches. While pharmacological interventions such as central nervous system stimulants (e.g., methylphenidate) and corticosteroids may provide short-term symptom relief, but long-term use may lead to side effects including metabolic disorders, immunosuppression (16, 17, 67). Consequently, non-pharmacological interventions have become the preferred approach in clinical practice. Although exercise therapy and psychological interventions are recommended as primary non-pharmacological measures, their adherence and efficacy vary significantly among individuals and may have limited effectiveness in patients with moderate-to-severe CRF (18, 19). Against the backdrop of cancer patients presenting with multiple symptoms and diverse needs, coupled with the advancement of precision medicine, further exploration of safe, effective, and personalized non-pharmacological therapies holds significant and urgent clinical value for the management of CRF (65).

In recent years, traditional acupuncture modalities such as manual acupuncture (MA), electroacupuncture (EA), and auricular acupuncture (AA) have been widely used to alleviate cancer-related symptoms due to their unique advantages of simplicity and minimal side effects (70, 20, 21). Traditional acupuncture exerts its therapeutic effects by stimulating specific acupoints to induce the sensation of “deqi”. In the field of CRF, acupuncture has been incorporated into the recommended treatment measures in the CRF guidelines established by the National Comprehensive Cancer Network (NCCN). However, due to methodological limitations in earlier research, such as inconsistencies in study quality, lack of standardized acupoint selection, and insufficient high-quality RCTs, the evidence strength within the guidelines remains low (22). With the further development of acupuncture in the CRF field in recent years, a large amount of new evidence has emerged to clarify and enhance the evidence strength of acupuncture treatment for CRF, suggesting that the current landscape of evidence may have undergone a transformation. Therefore, it is necessary to comprehensively update the evidence on the efficacy of acupuncture in the treatment of CRF and reassess its strength of evidence to provide reference information for clinical practice guidelines. Additionally, the current deficiencies of acupuncture in this field were identified during this process.

## 2 Materials and methods

This study has been registered in International prospective register of systematic reviews (PROSPERO, Registration Number: CRD42024603184) and reported according to the Preferred Reporting Items for Systematic Reviews and Meta-Analyses (PRISMA 2020) guidelines (23).

### 2.1 Search strategy

Two independent reviewers(HZ, ZP) conducted a comprehensive literature search in eight databases, including PubMed, Embase, Cochrane Library, Web of Science (WOS), China National Knowledge Infrastructure (CNKI), WeiPu (VIP), WanFang, and Chinese biomedical literature service system (SinoMed) from the inception of each database to April 2025. The search strategy employed a combination of medical subject headings (MeSH) and keywords. The specific MeSH terms included “acupuncture”, “electroacupuncture”, “auricular acupuncture”, “cancer”, “fatigue”, and “randomized controlled trial”. The search strategies were adjusted as necessary according to each database’s specific requirements. All search results were validated by a third reviewer(YH), with no language restrictions applied. The complete search strategies for each database are provided in **Supplementary Material**.

### 2.2 Inclusion criteria

The inclusion criteria were established based on the PICOS (participants, interventions, comparisons, outcomes, and study type) principle, as follows: (1) Participants: Patients in CRF status; (2) Intervention: Limited to traditional acupuncture types commonly used in oncology. Acupuncture treatment was administered as monotherapy or as an adjunct to standard therapy (e.g., acupuncture combined with standard care/conventional treatment); (3) Control group treatment: included sham acupuncture and conventional treatment, among others; (4) Outcome measures: The primary outcome was PFS, with additional outcomes including validated CRF-related assessment tools. (5) Study type: RCTs were prioritized due to their highest level of evidence. Details can be found in **Table 1**.

**Table 1:** Inclusion criteria.

PICOS	Inclusion criteria
Patient (P)	Patients diagnosed with CRF (No restriction on age, sex, country, cancer type, and stage)
Intervention (I)	①Acupuncture therapy as a sole treatment ②Acupuncture therapy as an adjunct to standard therapy (e.g., acupuncture therapy combined with standard care/conventional treatment)
Comparison (C)	Control group treatment, including sham acupuncture, standard care, and conventional treatment/no treatment.
Outcome (O)	Primary outcomes: Treatment effectiveness assessed by validated fatigue scales (e.g., Piper Fatigue Scale). Secondary outcomes: Other validated fatigue assessment scales.
Study type and others (S)	①Only randomized controlled trials (RCTs) are included. ②Language is unrestricted.

### 2.3 Exclusion criteria

Exclusion criteria are as follows: (1) Animal experiments, case reports, conference reports, abstracts, etc.; (2) Missing or inadequate outcome data; (3) Inaccessibility of full-text.

### 2.4 Literature screening

The retrieved literature was imported into Endnote X9.1. After deduplication, two researchers (HZ, ZP) independently reviewed the titles and abstracts of the literature based on the inclusion and exclusion criteria. They conducted a preliminary screening according to the inclusion criteria. Subsequently, for studies meeting the preliminary requirements, the full texts were read to finally determine the selected studies. For studies with incomplete outcome data, the researchers attempted to contact original authors via email or telephone to obtain necessary information. For studies with discrepancies, discussions were held with a third researcher (RL) to resolve the issues.

### 2.5 Data extraction

Data extraction was performed using pre-designed tables created in Microsoft Excel. The tables referred to the details of acupuncture interventions required by Standards for Reporting Interventions in Clinical Trials of Acupuncture (STRICTA) and combined with RCT-related characteristics were pre-designed and extracted using Microsoft Office Excel 2021 (24). The extracted data included general characteristics such as authors, publication year, country, cancer type, diagnostic criteria, patient age, outcome measures, etc.; specific acupuncture-related data such as acupoint selection and intervention duration; and statistical data including continuous variables presented as mean ± standard deviation (MD ± SD) related to outcome indicators, and dichotomous variables including sample size and event count (25).

### 2.6 Risk of bias assessment

The Cochrane Risk of Bias (ROB 2.0) tool was used to assess the risk of methodological bias. This tool evaluates five components (randomization process, deviations from intended interventions, missing outcome data, measurement of the outcome, and selection of the reported result). Based on the selections, each component is rated as some concern, low risk, or high risk. The overall risk of bias for the study is ultimately determined by aggregating the ratings for each component.

### 2.7 Statistical analysis

The meta-analysis was conducted using the “meta” package in R Studio. Effect sizes for continuous variables were expressed as mean difference(MD) or standardized mean difference (SMD), along with a 95% confidence interval (CI), based on the homogeneity of the measurement tools used in the different studies. Dichotomous data were represented using the relative risk (RR) and a 95% confidence interval (CI) at a significance level of 0.05. The magnitude of heterogeneity was quantified using the I^2^ statistic, where I^2^ <25% indicates low heterogeneity, I^2^ between 25-50% denotes moderate heterogeneity, and I^2^ >50% signifies high heterogeneity. Depending on the heterogeneity level, data were pooled using either a fixed-effects model or a random-effects model. Where at least three studies reported a given outcome, subgroup analyses were conducted based on acupuncture type and intervention duration to explore heterogeneity and effects of different acupuncture modalities. Sensitivity analyses were conducted using a leave-one-out approach to assess the robustness of the meta-analysis result. For outcomes with ten or more included studies, appropriate statistical methods were selected based on heterogeneity levels to evaluate the risk of publication bias (26).

## 3 Results

### 3.1 Study selection

**Figure 1:**
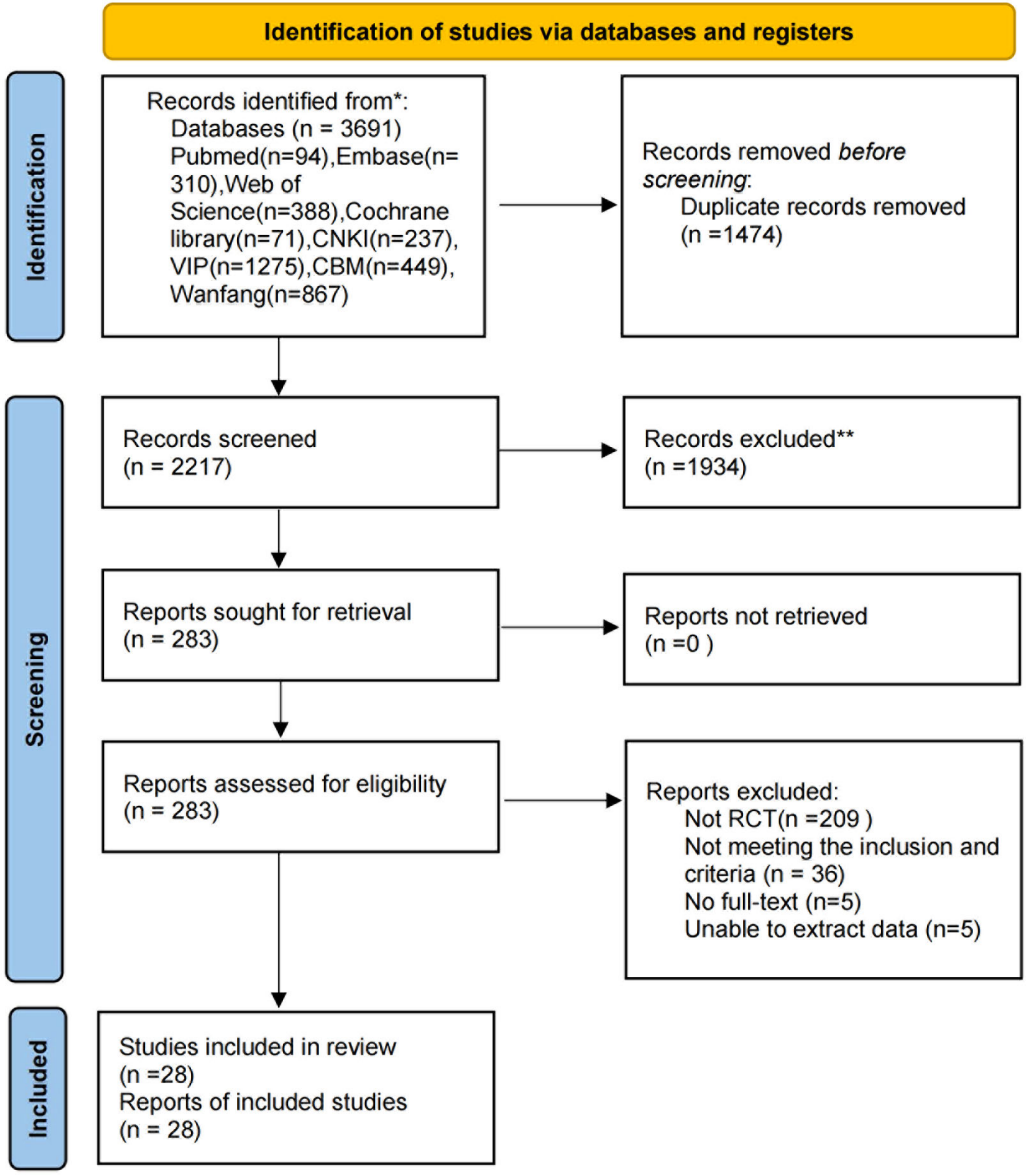
Flow diagram of screening process.

A preliminary search yielded 3,691 studies. Using the deduplication function, 1,474 studies were excluded. Subsequently, two independent reviewers screened titles and abstracts based on inclusion and exclusion criteria, initially excluding 1,934 studies, leaving 283 studies. Full-text reading was conducted for these 283 documents. Following full-text evaluation, 255 documents were excluded, resulting in the final inclusion of 28 studies. The flowchart of the study selection process is shown in **Figure 1**.

### 3.2 Study characteristics

The 28 studies included a total of 2,168 participants. The patients’ ages ranged from 49.77 (SD = 1.5) to 66.14 (SD = 2.4) years, with sample sizes varying from 21 to 162 across the studies. The studies were primarily conducted in China, with additional countries including the United Kingdom, Australia, the United States, and others. The types of cancer encompassed multiple categories such as colorectal cancer, breast cancer, and lung cancer. However, most studies did not restrict cancer types. The intervention group predominantly used MA, while the control group mostly employed standard therapy or sham acupuncture. Outcome measures included various tools such as BFI and PFS. Detailed characteristics are presented in **Table 2**.

**Table 2:** Study characteristic.

Author	Year	Country	Cancer type	Age (control/intervention)	Acupuncture type	Control	Existing time (sessions)	Acupuncture point	Outcome
Jun Wu (27)	2022	China	Lung	50.85 ± 7.24/50.78 ± 7.32	MA	UC	30mins (14)	CV17, CV6, CV12, ST36	CFS
Runxia Du (28)	2021	China	Any type	49.77 ± 1.5/54.32 ± 3.1	MA	UC	20mins (4)	GV20, ST36	BFI
Juan Shao (29)	2018	China	Lung	56.93 ± 9.79/58.50 ± 11.09	AA	UC	NR	Liver, Spleen, Kidney, Shenmen, Sympathetic points	PFS
Yuanyuan Zheng (30)	2023	China	Liver	59.9 ± 10.1/59.1 ± 9.9/58.4 ± 10.1	MA	TCM/MA+TCM	30mins (30)	CV6, CV4, ST36, BL23	PFS
Shufen Huang (31)	2022	China	Any type	56.10 ± 3.87/56.77 ± 2.19	AA	UC	1min (NR)	ST36	CFS
Xiushuang Li (32)	2016	China	Any type	62.2 ± 6.3/60.45 ± 7.71	MA	UC	30mins (14)	CV17, CV12, CV6, SP10, ST36, SJ5	MFI
Zeqing Lin (33)	2022	China	Colorectal	64.43 ± 11.22/64.67 ± 9.07	AA	UC	10mins (6)	Spleen, Shenmen, Heart, Subcortical, Kidney points	CFS
Juan Jiang (34)	2020	China	Any type	62.1 ± 2.1/62.5 ± 2.3	MA	UC	30mins (12)	CV17, CV12, CV6, SP10, ST36, SJ5	CFS
Peng Qing (35)	2020	China	Any type	59 ± 4/60 ± 3	MA	UC	15mins (20)	GV20, CV4, CV6, GB20, ST36, SP6	FACT-F
Longjiao Tao (36)	2020	China	Any type	61.3 ± 10.03/63.91 ± 7.92	MA	UC	30mins (7)	CV4, ST36, SP6, LR4, KI3, HT7, PC6 GB12	BFI
Hong Pan (37)	2018	China	Breast	52.03 ± 6.55	MA	SA	NR (8)	GV20, CV6, ST36	PFS
Ya Su (38)	2016	China	Any type	62 ± 6/60 ± 11	MA	UC	30mins (14)	KI3, GB39, ST36, SP10, CV6, CV4	PFS
Mingwei Yu (39)	2017	China	Breast	51.4 ± 8.4/50.2 ± 8	MA	SA	NR (8)	GV20, CV4, CV6, ST36, SP6	PFS
Shengyun Li (40)	2018	China	Gynaecological	66.04 ± 2.30/66.14 ± 2.40	MA	UC	20mins (9)	CV12, ST36, PC6, SP6, CV6, CV4, BL23	BFI
Liyuan Guo (41)	2014	China	Gynaecological	53 ± 6.2/51 ± 5.5/52 ± 3.7	MA	UC/TCM	20mins (9)	CV12, PC6, ST36, SP6, CV6, CV4, BL23	BFI
Xiuting Du (42)	2021	China	Colorectal	60.96 ± 11.42/61.04 ± 12.21	MA	UC	30mins (NR)	CV6, CV4, ST36, BL23	PFS
Li Liu (43)	2021	China	Any type	61.02 ± 5.88/61.58 ± 6.13	MA	UC	30mins (6)	GV29, GV20, PC6, LI4, SP6, ST36, SP9, GB34, KI3, LR3	PFS
Xiuting Du (44)	2024	China	Intestinal	61.83 ± 10.55/55.62 ± 12.04	MA	UC	30mins (NR)	CV6, CV4, ST36	PFS
Alexander Molassiotis (45)	2007	UK	Any type	53.4 ± 13.1	MA	SA/acupressure	20mins (6)	ST36, SP6, LI4	MFI
Weidong Lu (46)	2012	MA	Ovarian	50 ± 9.9/50.8 ± 10.6	EA	SA	30mins (12)	SP10, ST36, SP6, K3, LR3, GV20, LI11, LI4, PC6	EORTC-QLQ-C30
Jun J.Mao (47)	2014	USA	Breast	57.5 ± 10.1/60.9 ± 6.5/60.6 ± 8.2	MA	SA/WLC	20mins (10)	Around the joint with the most pain	BFI
Jinxia Li (48)	2023	China	Breast	52.91 ± 11.89/52.46 ± 14.01	MA	UC	30mins (20)	GV20, GV29, LR3, LI4, ST36, SP6, HT7	MFI
Caroline Smith (49)	2013	Australia	Breast	58 ± 7.5/53 ± 12.5/55 ± 8.8	MA	SA/WLC	30mins (9)	KI3, KI²7, ST36, SP6, CV4, CV6	BFI
Gary Deng (50)	2013	USA	Any type	NR	MA	SA	20mins (6)	CV6, CV4, KI3, ST36, SP6, LI11, HT6	BFI
Chien-shan Cheng (51)	2017	China	Lung	62 ± 4.3/58 ± 5.2	MA	SA	45mins (8)	LI4, CV6, KI3, ST36, SP6	BFI
Melanie D. Höxtermann (52)	2021	Germany	Breast	54.8 ± 8.3/56.58 ± 7.9	AA	psychoeducation	20mins (10)	NR	FACT-F
Yali Gao (53)	2025	China	Cervical	51.52 ± 6.85/50.34 ± 6.12	EA	UC	30mins (18)	CV12, GV20, CV6, CV4, GV4, CV3, GV3, GV2	PFS
Xin Chen (54)	2025	China	Breast	48.80 ± 21.37/49.60 ± 25.28/48.03 ± 22.47	MA	SA/WLC	30mins (12)	SP4, PC6, LU7, LI6, BL62, SI3, GB41, TE5	PFS

MA, manual acupuncture; EA, electroacupuncture; UC, usual care; SA, sham acupuncture; WLC, wait list control; TCM, traditional Chinese medicine; NR, Not report; AA, auricular acupuncture; MFI, multidimensional fatigue inventory; BFI, brief fatigue inventory; FACT-F, functional assessment of cancer therapy-fatigue.

### 3.3 Study quality

**Figure 2:**
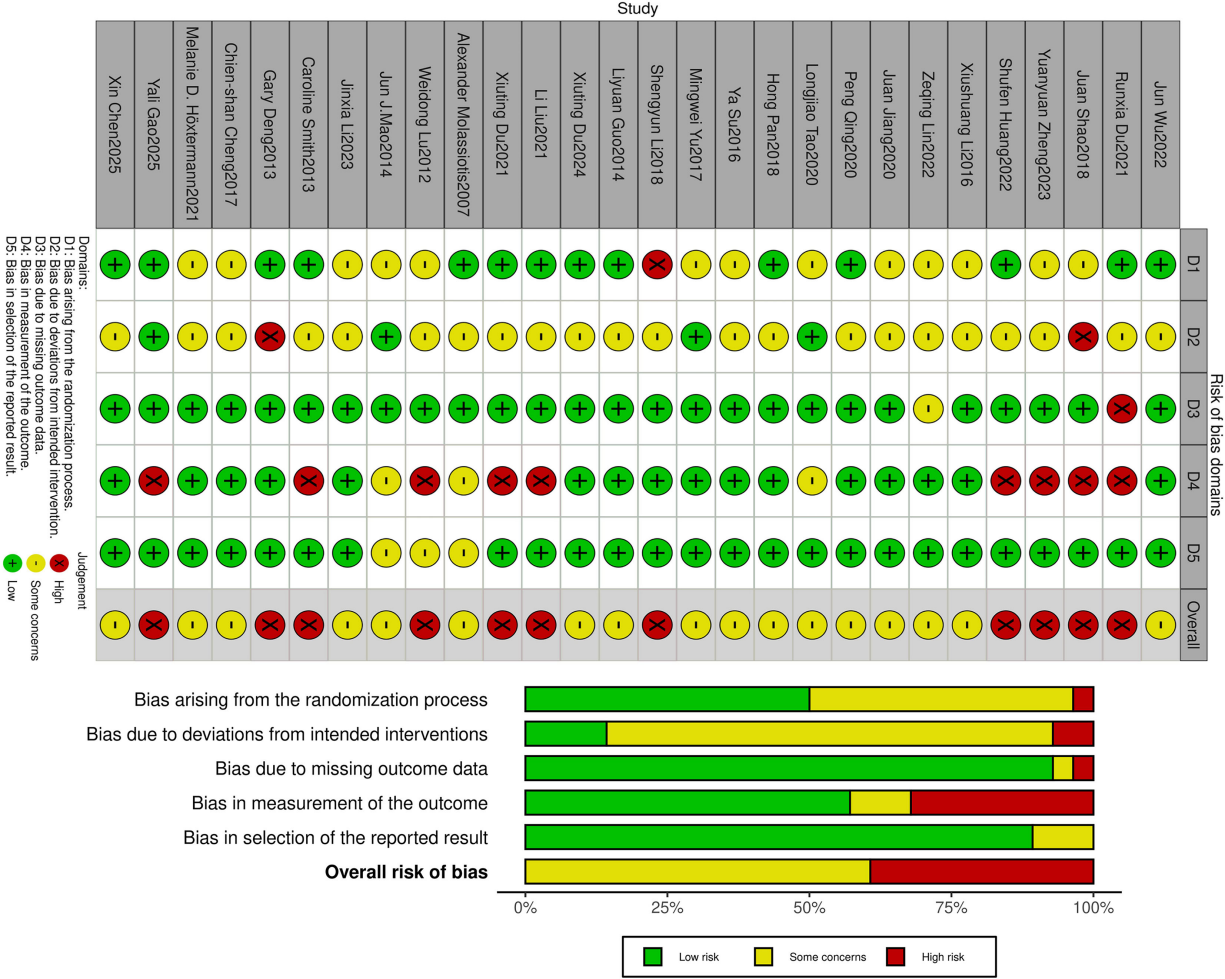
Risk of bias.

The risk of bias assessment results showed that 11 studies were classified as having “high risk of bias”, 17 studies raised “some concerns”, and no study was rated as having a “low risk of bias”. In the assessment of randomization bias, most studies were downgraded due to unclear reporting of randomization methods and allocation concealment. For intervention bias related to blinding, the majority were downgraded due to inadequate blinding implementation. Because it is difficult to blind the acupuncturists. Regarding outcome measurement bias, most studies were downgraded because the assessment tools used were not validated. Specific bias risk results are shown in **Figure 2**.

### 3.4 Results of the meta-analysis

#### 3.4.1 PFS

**Figure 3:**
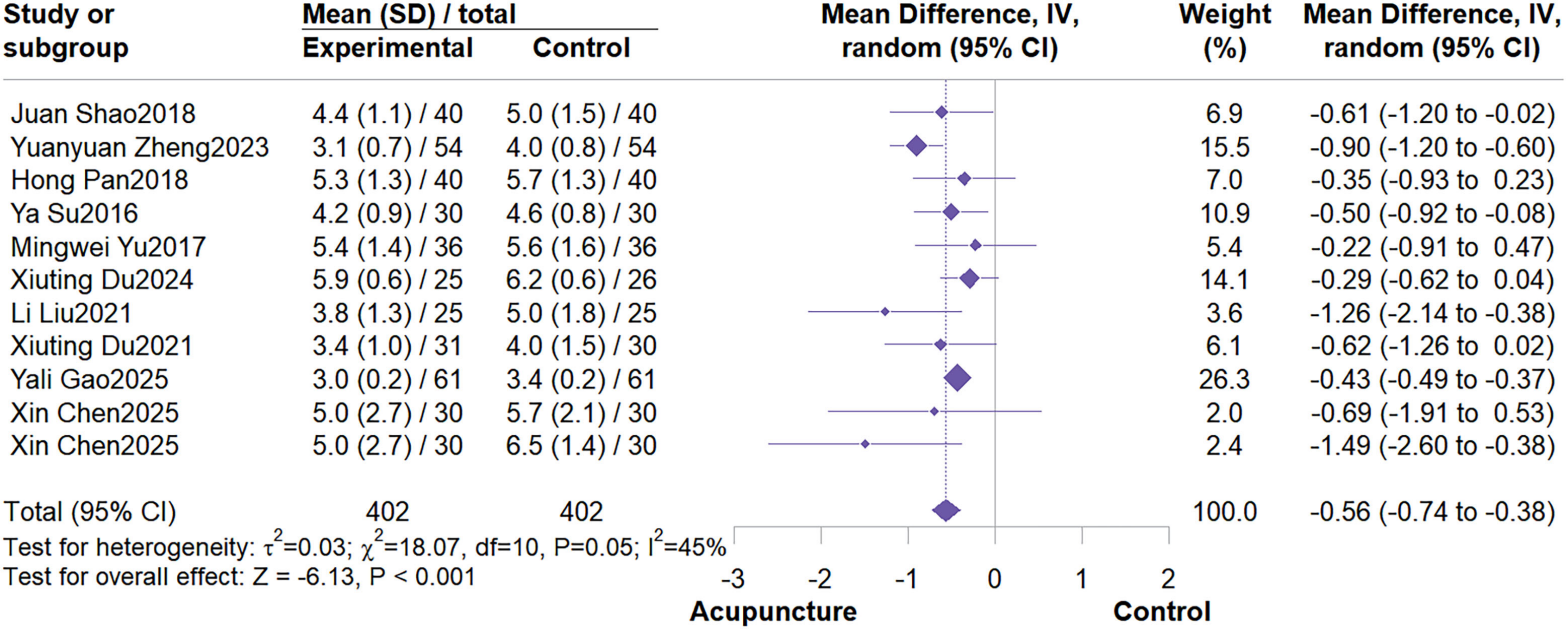
Forest plot of PFS.

**Figure 4:**
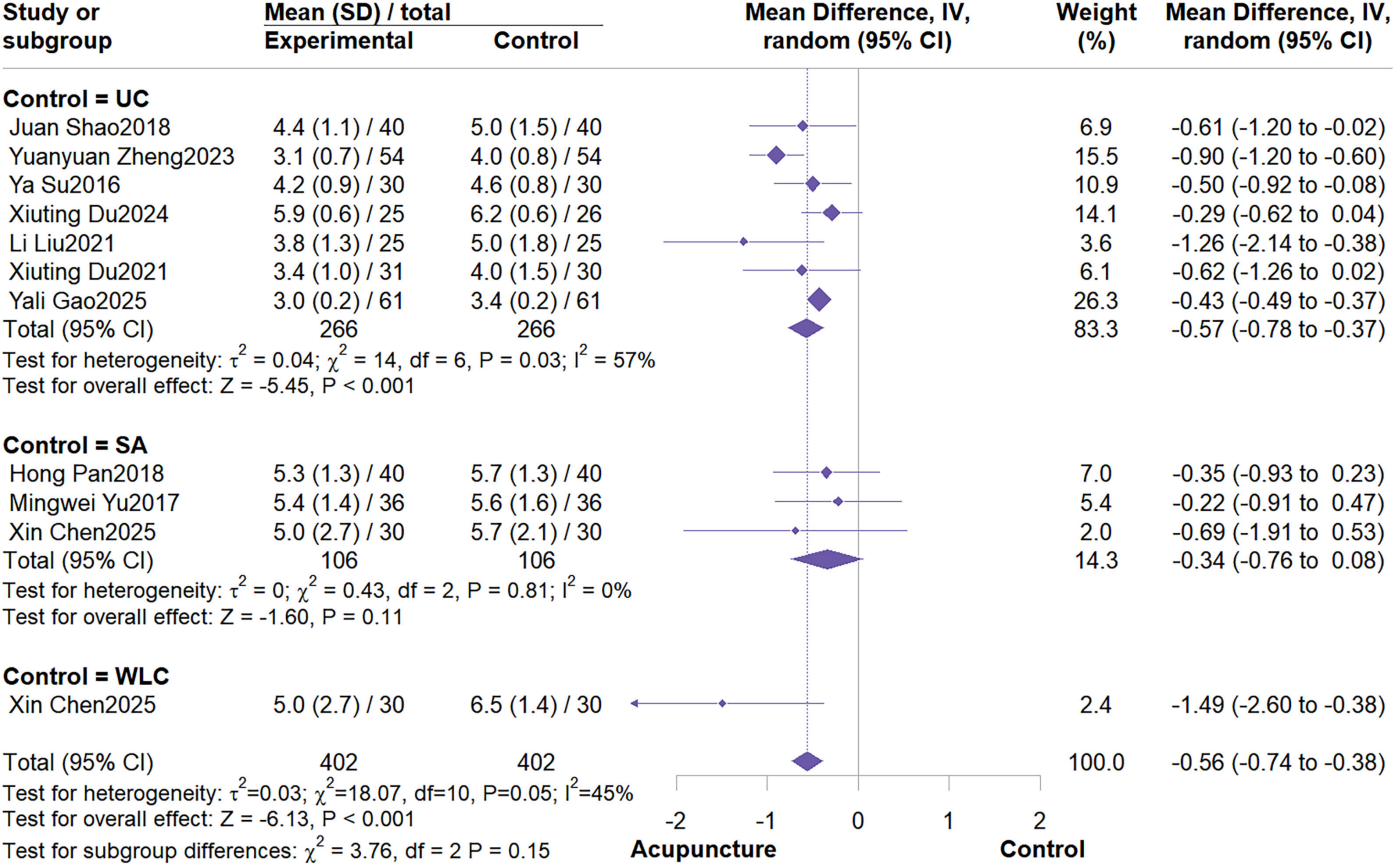
Subgroup analysis by control group type.

**Figure 5:**
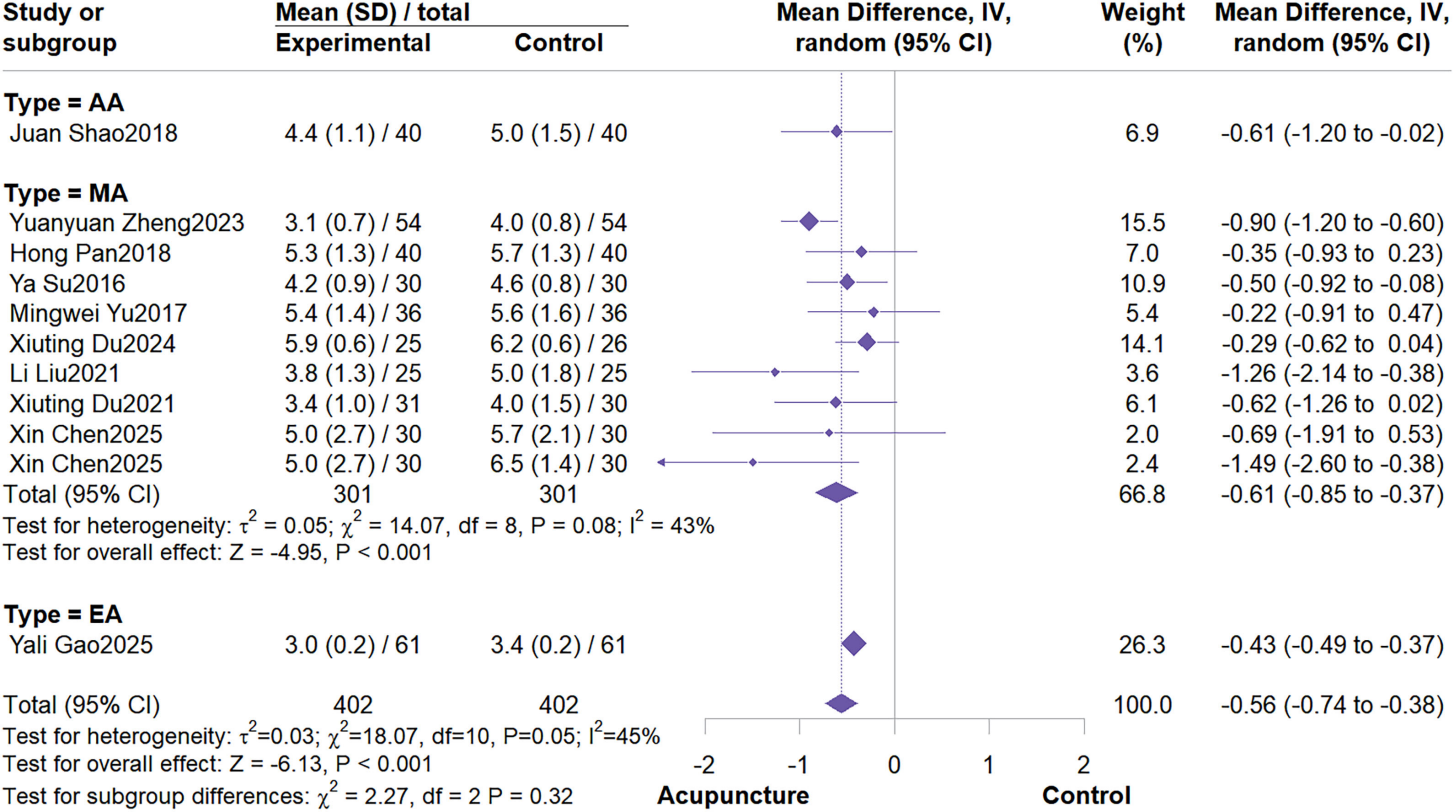
Subgroup analysis by acupuncture type.

This meta-analysis included 10 RCTs with a total sample size of 804 patients. The meta-analysis demonstrated that acupuncture effectively reduced PFS scores (MD = -0.56; 95% CI: -0.74 to -0.38, I^2^ = 45%, P<0.001) (**Figure 3**). Further subgroup analyses based on control group type and acupuncture type indicated no potential sources of heterogeneity (**Figure 4**; **Figure 5**). Subgroup analysis by control group revealed that acupuncture significantly reduced PFS scores compared to conventional treatment or wait list control, but failed to reduce PFS scores when compared to sham acupuncture (MD = -0.34; 95% CI: -0.76 to 0.08; I^2^ = 0%, P = 0.11). Subgroup analysis by acupuncture type revealed that MA demonstrated significantly greater efficacy in reducing PFS scores compared to other acupuncture types (MD = -0.61; 95% CI: -0.85 to -0.37; I^2^ = 43%, P < 0.01). Sensitivity analysis confirmed the robustness of these results (**Supplementary Material**, **Supplementary Figure S1**).

#### 3.4.2 Brief fatigue inventory

**Figure 6:**
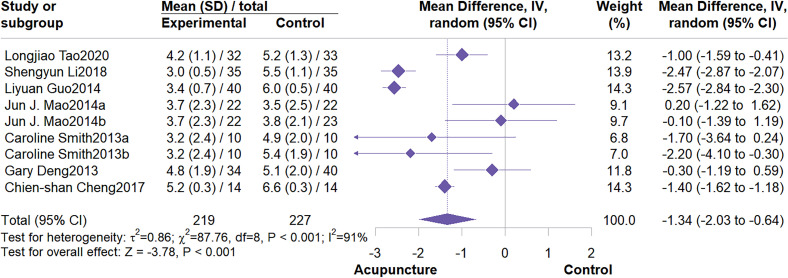
Forest plot of BFI.

**Figure 7:**
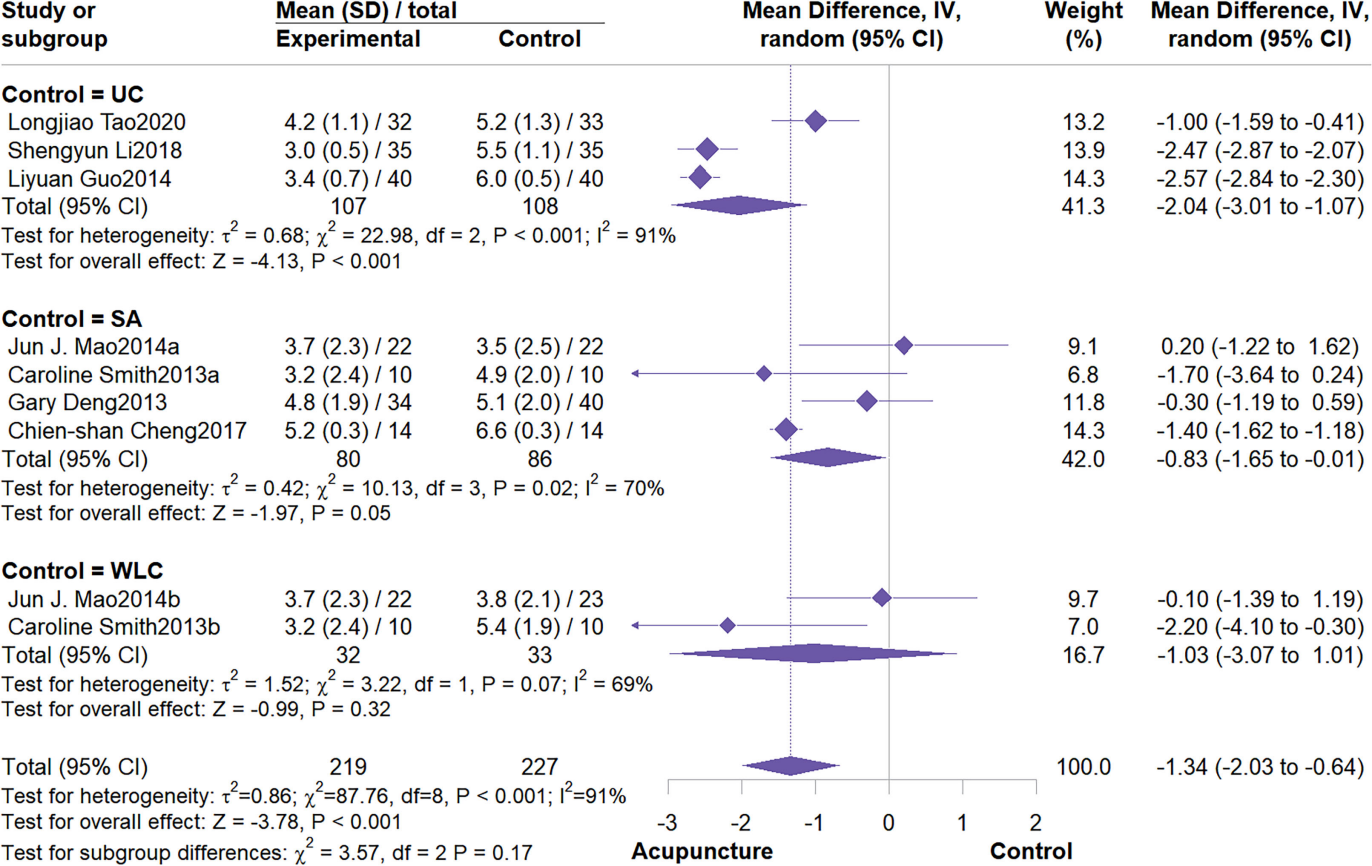
Subgroup analysis by control group type.

**Figure 8:**
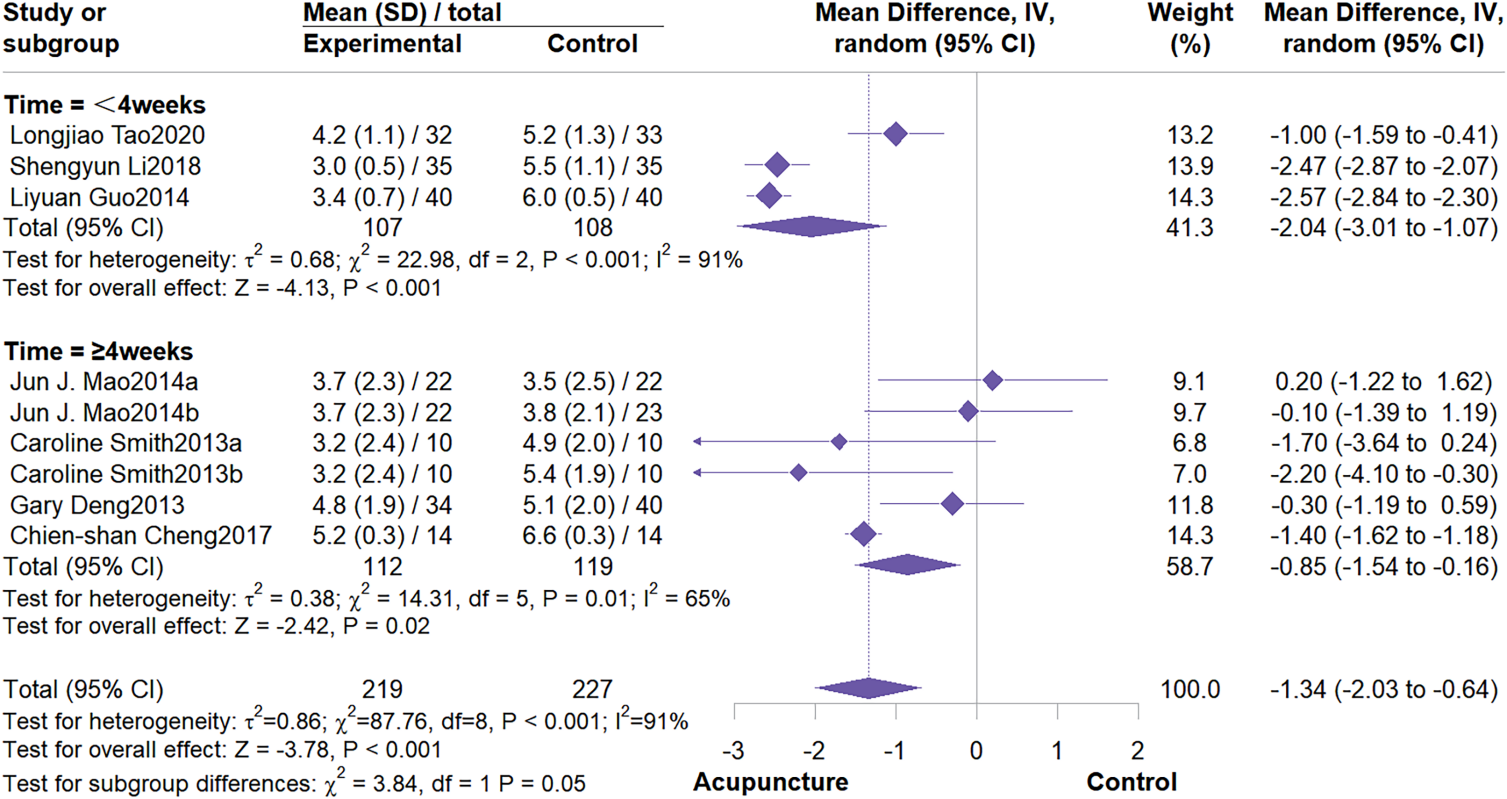
Subgroup analysis by intervention duration.

This meta-analysis included 7 studies involving 446 patients. The meta-analysis demonstrated that acupuncture significantly outperformed the control group in reducing BFI fatigue scores (MD = -1.34, 95% CI: -2.03 to -0.64, I^2^ = 91%, P < 0.001) (**Figure 6**). Subgroup analysis indicated that control group type and intervention duration were not potential sources of heterogeneity. Furthermore, subgroup analysis demonstrated that acupuncture effectively alleviate fatigue symptoms, particularly when compared to usual care (MD = -2.04, 95% CI: -3.01 to -1.07; I^2^ = 91%, P < 0.001). When compared to wait list control, the effect was not statistically significant (**Figure 7**). Regarding intervention duration, subgroup analysis suggested that short-term (<4 weeks) interventions were significantly more effective than long-term (≥4 weeks) interventions (**Figure 8**). Sensitivity analysis confirmed the reliability of the results (**Supplementary Material**, **Supplementary Figure S2**).

#### 3.4.3 Chalder fatigue scale

**Figure 9:**
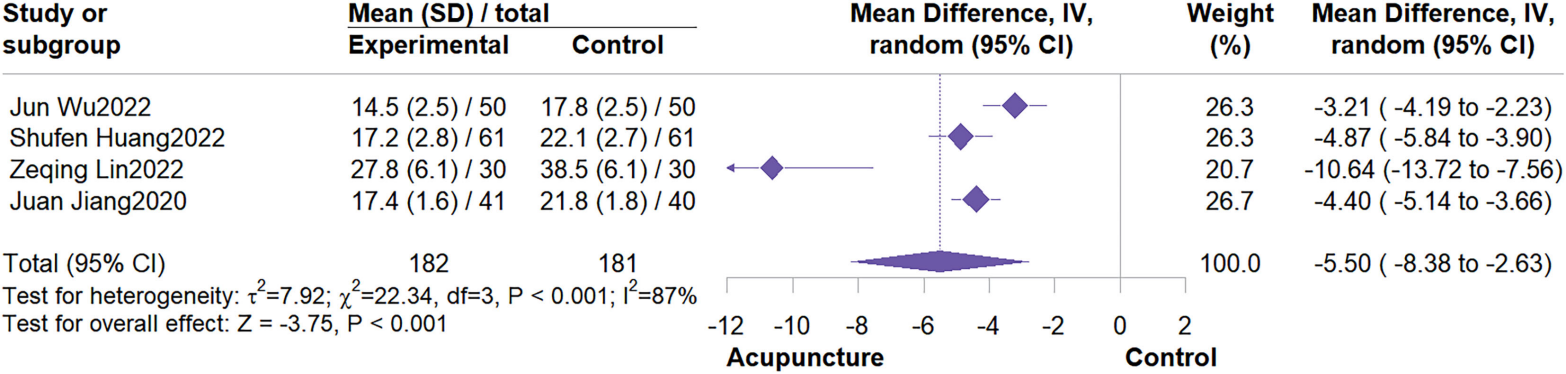
Forest plot of CFS.

**Figure 10:**
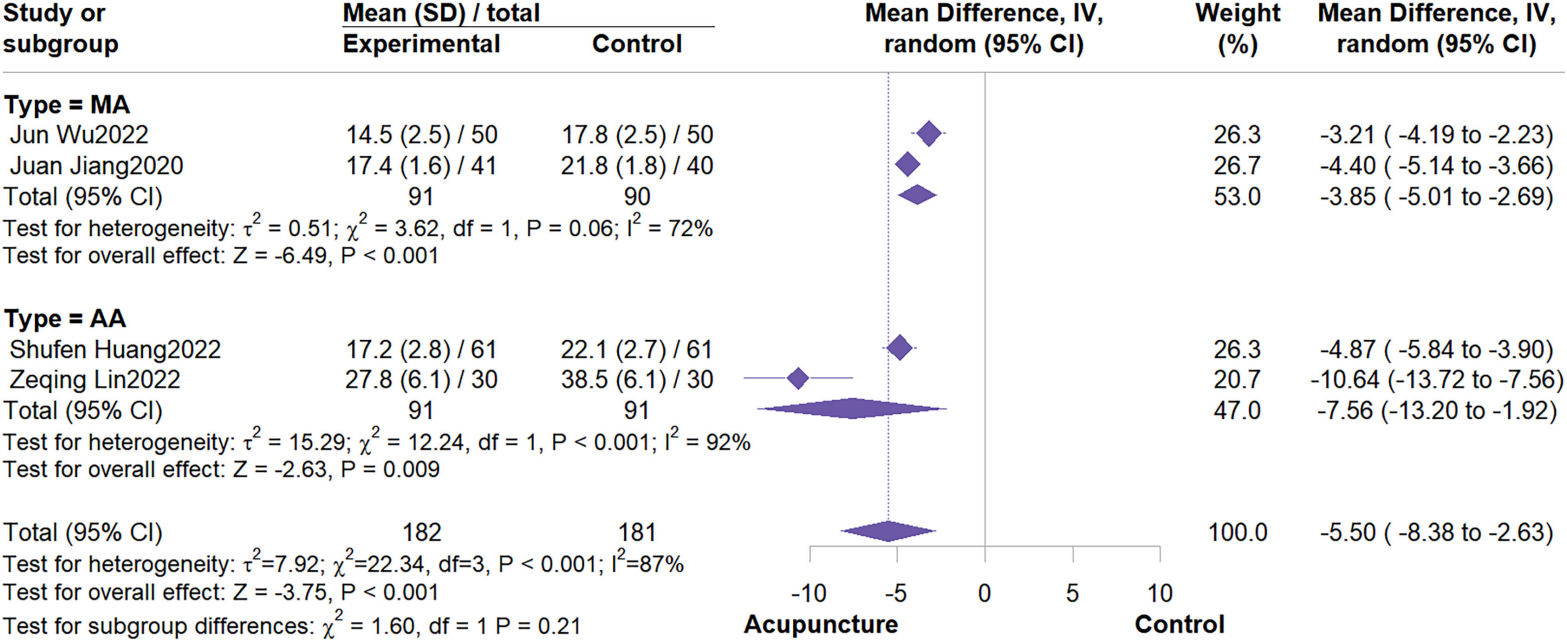
Subgroup analysis by acupuncture type.

4 studies involving 363 participants were included. The meta-analysis demonstrated that acupuncture effectively improved fatigue severity compared to control groups (MD = -5.50, 95% CI: -8.38 to -2.63, I^2^ = 87%, P < 0.01)(**Figure 9**). Subgroup analysis revealed that acupuncture type was not a potential source of heterogeneity. Results also indicated that both MA and AA showed significant efficacy in improving fatigue severity(**Figure 10**). Sensitivity analysis showed that excluding individual studies altered the effect estimate, suggesting results for this outcome should be interpreted cautiously (**Supplementary Material**, **Supplementary Figures S3**).

#### 3.4.4 Multidimensional fatigue inventory

**Figure 11:**
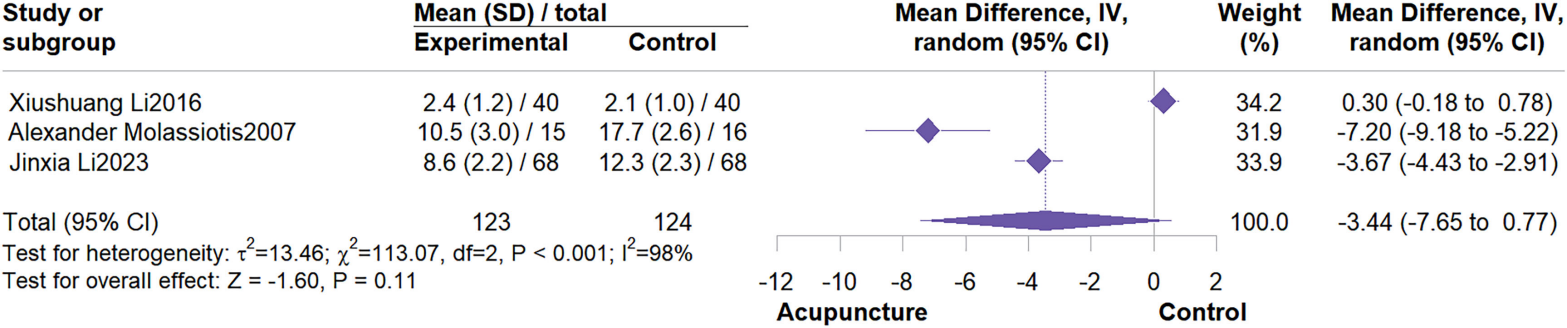
Forest plot of MFI.

3 studies were included in the meta-analysis for this outcome. Results showed that acupuncture did not improve MFI scores compared with the control group (MD = -3.44, 95% CI: -7.65 to 0.77; I^2^ = 98%, P = 0.11) (**Figure 11**). Sensitivity analysis also suggested cautious interpretation of this outcome (**Supplementary Material**, **Supplementary Figure S4**).

### 3.5 Publication bias

**Figure 12:**
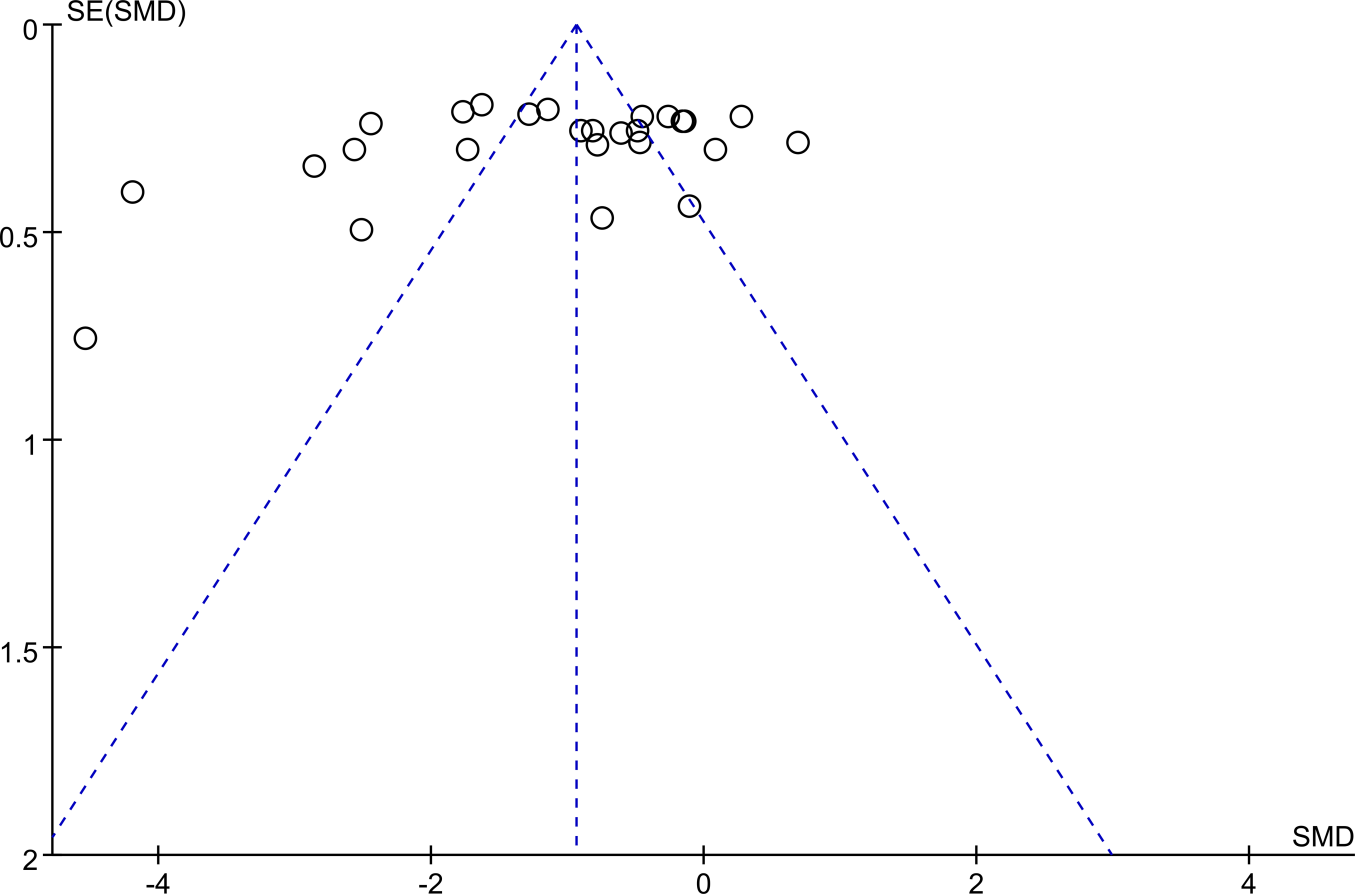
The funnel plot related to hot flash-related symptom scale.

As shown in **Figure 12**, The uneven distribution of scatter points suggests the possibility of publication bias.

### 3.6 GRADE evidence assessment

The GRADE system was used to evaluate the quality of evidence from 16 meta-analyses. We primarily analysed outcomes from two pairs of comparisons: MA versus usual care and MA versus sham acupuncture. Results showed that in the MA versus usual care comparison, only four studies using CFS as an outcome were rated “moderate quality”; the remainder were rated “very low quality”. In the MA versus sham acupuncture comparison, all outcome measures were rated as “low quality”. Detailed information is provided in the **Supplementary Material** (**Supplementary Tables S1**, **S2**).

## 4 Discussion

### 4.1 Summary of key findings

This meta-analysis included 28 randomized controlled trials (RCTs) involving 2,168 patients to evaluate the efficacy of traditional acupuncture in treating cancer-related fatigue (CRF). Overall, the meta-analysis indicated that traditional acupuncture shows potential benefits in improving CRF symptoms across most outcome measures compared to control groups; however, given the heterogeneity between studies and the quality of evidence, these results should be interpreted with caution. We conducted subgroup analyses based on acupuncture type, control group type, and intervention duration to explore and identify sources of heterogeneity. Subgroup analysis results showed that MA may possesses unique advantages in improving CRF, which is consistent with previous research findings (55). Moreover, among the primary outcome measures in this study, acupuncture did not show a statistically significant difference compared to sham acupuncture (MD=-0.34, 95% CI: -0.76 to 0.08, P = 0.11). Furthermore, this meta-analysis revealed a certain time-dependent relationship between the efficacy of acupuncture and the duration of the intervention. Short-term (<4 weeks) acupuncture appears to produce better therapeutic effects on CRF symptoms, while the efficacy may diminish as the intervention duration extends (≥4 weeks).This finding complements previous meta-analyses that focused solely on overall efficacy. However, in the risk of bias assessment, most included studies were judged to have a high risk of bias. Therefore, although the meta-analysis results suggest that acupuncture can effectively treat CRF, given the low quality of the included studies, more rigorously designed, high-quality randomized controlled trials are still needed to improve the certainty of the evidence and confirm the effectiveness of acupuncture.

### 4.2 Clinical implications

Currently, non-pharmacological therapies are the preferred treatment approach for CRF in both clinical guidelines and real-world practice. Although exercise therapy is highly recommended (68, 56), its implementation faces significant barriers in clinical practice due to the highly complex individual circumstances of cancer patients, including contraindications such as bone metastases and fall risk (57).In such cases, exercise therapy encounters notable obstacles in clinical application. Acupuncture, as a therapy with both universality and high personalization, offers a novel treatment option for CRF (55). Its universality is reflected in the rarity of contraindications (21), enabling it to be applied to a broad population. Personalization is demonstrated by its ability to target different acupoints for the treatment of specific symptoms in distinct patient groups. Therefore, during the early stages of advocating the concept of treating CRF, numerous studies had already demonstrated the efficacy of acupuncture in treating CRF, which were subsequently incorporated into the NCCN clinical guidelines. However, the evidence strength remained constrained due to the prevalent limitations and biases in early evidence-based research on acupuncture. Although numerous studies have emerged in recent years aiming to overcome these challenges, it is regrettable that, based on the risk of bias findings from this study, the issue remains unresolved, with low-quality research still constituting the majority. However, this meta-analysis revealed an intriguing finding: MA demonstrated superior efficacy in alleviating CRF symptoms compared to other acupuncture modalities. Although sensitivity analyses for certain outcomes showed instability, the results still provide valuable clinical insights, suggesting that MA may be more appropriately considered for CRF patients with individualized conditions. Simultaneously, there is an urgent need for further studies to explore the distinct therapeutic advantages of MA and other acupuncture modalities in CRF management. In summary, current clinical decision-making regarding acupuncture for CRF should adhere to the recommendations of the NCCN guidelines, primarily applying acupuncture to patients in the active treatment phase. When applicable, MA protocols may be prioritized.

### 4.3 Implications for future research

In the subgroup analysis of the primary outcome measure (PFS) categorized by control group type, we found that acupuncture failed to show a significant difference compared to sham acupuncture. To investigate this issue, we conducted a structured summary of the sham acupuncture protocols used in the studies (**Supplementary Material**, **Supplementary Table S3**). The analysis revealed that the sham acupuncture in the three included studies all employed superficial needling at non-acupuncture points. Previous research has indicated that superficial needling at non-points is not entirely “inert”, It inherently possesses therapeutic effects, as research on sleep disorders indicates that non-acupoint shallow needling can effectively improve the Pittsburgh Sleep Quality Index (PSQI) score after treatment (58, 69, 59). Based on the above, our failure to observe an advantage of verum acupuncture over sham acupuncture may be due to the specific therapeutic effects of invasive sham acupuncture devices, while the natural progression of the disease cannot be ruled out. In contrast, non-penetrating sham acupuncture devices have been shown to minimize this confounding effect. Consequently, future research should focus more on non-penetrating sham acupuncture, high-quality large-sample studies, and the precise quantification of acupuncture’s actual efficacy. However, considering the diversity of sham acupuncture and its potential impact on effect size estimation, as well as the possibility of heterogeneity, placebo effects, or contextual effects, we remain cautious in drawing this inference. Additionally, this meta-analysis revealed that short-term acupuncture courses may demonstrate greater efficacy for CRF treatment compared to long-term courses. However, some studies suggest that acupuncture may require repeated stimulation to achieve functional reorganization in fatigue-related brain regions (such as the default mode network and anterior cingulate cortex), implying that longer treatment courses might be more effective (60). Therefore, such findings should be interpreted with caution, as they may reflect small-scale study effects, publication bias, or other confounding factors. To address this, future studies are expected to include comparative trials of short-and long-course acupuncture treatments for CRF to clarify this issue.

Currently, at the mechanistic level, both animal experiments and clinical studies collectively indicate that acupuncture may improve CRF through multiple pathways. These include regulating the HPA axis function, modulating pro-inflammatory factor levels, and promoting energy metabolism. Among these studies, most employed Zusanli (ST36) as the core acupoint, which aligns with the acupoint characteristics in the included studies. However, when ST36 is used as the core point, it remains unclear which acupoint can maximize synergistic effects. Some studies suggest that the synergistic mechanism between ST36 and Guanyuan (CV4) can significantly modulate immune function (61), such as enhancing natural killer cell activity, regulating the proportion of T lymphocyte subsets, and reducing levels of pro-inflammatory cytokines (e.g., IL-1β, IL-6, TNF-α), thereby alleviating key pathological aspects of CRF (35).However, both the current mechanism of acupuncture effects and the optimal combination of acupoints for CRF treatment efficacy or their underlying mechanisms require further elucidation. We anticipate that future research will address this issue.

Further analysis of the overall characteristics of the included studies revealed that the tools used to assess CRF were overly subjective, with no objective evaluation tools employed. Future research could explore the use of wearable devices (e.g., accelerometers) to monitor daily activity levels or detect inflammatory markers (e.g., IL-6, CRP) to provide more objective evidence of treatment efficacy. In this meta-analysis, most included studies provided overly simplistic descriptions of the randomization process without specifying the exact randomization methods. Future studies should ensure complete and accurate reporting of the randomization procedures. Furthermore, due to the unique nature of acupuncture practice, it is challenging for acupuncturists to implement effective blinding methods. Therefore, it is particularly crucial to ensure consistent blinding for both participants and evaluators throughout the study process. However, in the studies included in this meta-analysis, sham acupuncture, as the most commonly used blinding method in acupuncture research, has shown significant limitations in its application. Its current role as a “placebo” in the control group remains controversial (62, 63).Future research could validate the physiological inertness of sham acupuncture by incorporating functional magnetic resonance imaging (fMRI) or peripheral biomarkers to reduce interference from placebo effects. Regarding the mechanisms of acupuncture in treating CRF, metabolomics, genomics, and proteomics can explore potential pathways through which acupuncture modulates energy metabolism and immune function. Simultaneously, fMRI or positron emission tomography (PET) can be employed to study the effects of acupuncture on the default mode network (DMN) and fatigue-related brain regions (e.g., anterior cingulate cortex). In terms of clinical trial design, future research should provide detailed reports on acupoint selection and stimulation parameters (e.g., electroacupuncture frequency, needle retention time) to establish standardized acupuncture protocols (acupoints, stimulation parameters, treatment duration) for clinical implementation. In summary, research on acupuncture for CRF urgently requires improvements in these areas. High-quality RCTs are required to strengthen the evidence base and guide the development of clinical practice guidelines.

### 4.4 Research limitations and strengths

#### 4.4.1 Limitations

Firstly, this meta-analysis demonstrated that acupuncture showed a statistically significant improvement in the primary outcome measure of PFS with a MD of-0.56 (95% CI: -0.74 to-0.38). However, the actual clinical value of this difference requires further evaluation. Studies in patients with breast cancer have suggested that a 2-point decrease in the PFS total score is perceived as a meaningful clinical improvement (64), whereas the magnitude of change observed in this study was relatively limited. Furthermore, no consensus has yet been reached regarding the minimal clinically important difference (MCID) for the PFS that reflects clinically meaningful improvement, which may affect comparability across studies. Future research should clarify relevant evaluation thresholds and incorporate individual response-based outcome measures to more comprehensively reflect the actual efficacy of interventions.

Secondly, the literature included in this study has certain limitations, specifically characterized by a high risk of bias and small sample sizes. These factors may affect the accuracy and reliability of the assessment of acupuncture’s efficacy. Furthermore, although subgroup analyses were conducted to explore heterogeneity, significant differences persisted among the included studies. This may stem from several factors: this study investigated the efficacy of traditional acupuncture for pan-cancer fatigue and included more recently published RCTs, which introduced more complex clinical heterogeneity. However, the underlying mechanisms of fatigue in different states (e.g., chemotherapy-induced fatigue, recovery-phase fatigue, and end-stage cancer fatigue) may differ, involving variations in inflammatory response levels, neuroendocrine regulation, and psychological factors. These differences could lead to inconsistent responses to acupuncture interventions. Simultaneously, different cancer types and treatment stages may also influence pathophysiology and treatment response. Due to the insufficient information reported in existing RCTs on CRF, it is difficult to conduct in-depth subgroup analyses for different cancers and states. Therefore, the study’s conclusions should be interpreted with caution.

#### 4.4.2 Strengths

However, this study possesses some advantages. We systematically integrated a large amount of independent outcome data to comprehensively evaluate the efficacy of acupuncture on multiple indicators of CRF. Previous systematic reviews have predominantly focused on specific cancer populations. For instance, a meta-analysis demonstrated that acupuncture exhibits a modest effect in alleviating breast CRF, with low interstudy heterogeneity (66). In contrast, this study adopted a pan-cancer inclusion strategy, expanding the research scope. Although higher levels of heterogeneity were observed, the findings were generally consistent with previous studies in terms of therapeutic efficacy. By integrating evidence from a broader population, this study further supplements the evidence regarding the efficacy of acupuncture in treating CRF. Subgroup analyses further revealed the specific effects of different acupuncture therapies on CRF. Although the overall quality of included trials requires cautious interpretation, this review still provides a crucial supplement to the existing evidence system.

## 5 Conclusion

This meta-analysis suggests that acupuncture may alleviate CRF, particularly in patients who have completed anti-cancer treatment but still suffer from chronic fatigue. It shows potential for some short-term symptom improvement compared to standard care. It shows potential for some short-term symptom improvement compared to standard care. However, due to methodological limitations in the included studies, significant heterogeneity, and unclear efficacy when comparing acupuncture to sham acupuncture, the certainty of the current evidence is low to very low. Therefore, the definitive efficacy of acupuncture as an adjuvant therapy for CRF remains to be further confirmed through high-quality randomized controlled trials with more rigorous designs and larger sample sizes. Future research should also explore and incorporate clinical significance thresholds for CRF-related scales and develop standardized treatment protocols to promote the standardized application of acupuncture in supportive oncology care.

## Data Availability

The original contributions presented in the study are included in the article/**Supplementary Material**. Further inquiries can be directed to the corresponding authors.
