# Supplementary material for: Effectiveness of acupuncture for cancer-related fatigue: a systematic reviews and meta-analysis
Source: Front Oncol. 2026 May 5;16:1790238. doi: 10.3389/fonc.2026.1790238 (PMC13183552; doi:10.3389/fonc.2026.1790238)
Supplement: Supplementary file 1 [file DataSheet1.docx]

Supplementary Material

# Supplementary Data

The Search strategy for PubMed.

| No | Search history |
| --- | --- |
| #1 | "Acupuncture"[Mesh] OR "Acupuncture Therapy"[Mesh] OR "Acupuncture, Ear"[Mesh] |
| #2 | "Electroacupuncture"[Mesh] |
| #3 | #1 OR #2 |
| #4 | (((((((((((((((((Pharmacopuncture[Title/Abstract]) OR (Acupuncture Treatment[Title/Abstract])) OR (Acupuncture Treatments[Title/Abstract])) OR (Treatment, Acupuncture[Title/Abstract])) OR (Therapy, Acupuncture[Title/Abstract])) OR (Pharmacoacupuncture Treatment[Title/Abstract])) OR (Treatment, Pharmacoacupuncture[Title/Abstract])) OR (Pharmacoacupuncture Therapy[Title/Abstract])) OR (Therapy, Pharmacoacupuncture[Title/Abstract])) OR (Acupotomy[Title/Abstract])) OR (Acupotomies[Title/Abstract])) OR (Acupunctures, Ear[Title/Abstract])) OR (Ear Acupunctures[Title/Abstract])) OR (Acupuncture, Auricular[Title/Abstract])) OR (Acupunctures, Auricular[Title/Abstract])) OR (Auricular Acupunctures[Title/Abstract])) OR (Auricular Acupuncture[Title/Abstract])) OR (Ear Acupuncture[Title/Abstract]) |
| #5 | #3 OR #4 |
| #6 | "Fatigue"[Mesh] |
| #7 | ((Lassitude[Title/Abstract]) OR (Tiredness[Title/Abstract])) OR (Weary[Title/Abstract]) |
| #8 | #6 OR #7 |
| #9 | "Neoplasms"[Mesh] |
| #10 | ((((((((((((((((Tumors[Title/Abstract]) OR (Neoplasia[Title/Abstract])) OR (Neoplasias[Title/Abstract])) OR (Neoplasm[Title/Abstract])) OR (Tumor[Title/Abstract])) OR (Cancer[Title/Abstract])) OR (Cancers[Title/Abstract])) OR (Malignant Neoplasm[Title/Abstract])) OR (Malignancy[Title/Abstract])) OR (Malignancies[Title/Abstract])) OR (Malignant Neoplasms[Title/Abstract])) OR (Neoplasm, Malignant[Title/Abstract])) OR (Neoplasms, Malignant[Title/Abstract])) OR (Benign Neoplasms[Title/Abstract])) OR (Neoplasms, Benign[Title/Abstract])) OR (Neoplasm, Benign[Title/Abstract])) OR (Benign Neoplasm[Title/Abstract]) |
| #11 | #9 OR #10 |
| #12 | "Randomized Controlled Trial" [Publication Type] OR "Randomized Controlled Trials as Topic"[Mesh] OR "Controlled Clinical Trial" [Publication Type] |
| #13 | ((((((randomized controlled trial[Title/Abstract]) OR (controlled clinical trial[Title/Abstract])) OR (clinical trials as topic[Title/Abstract])) OR (trial[Title/Abstract])) OR (random*[Title/Abstract])) OR (placebo*[Title/Abstract])) OR (search[Title/Abstract]) |
| #14 | #12 OR #13 |
| #15 | #5 AND #8 AND #11 AND #14 |

The Search strategy for Embase.

| No | Search history |
| --- | --- |
| #1 | 'acupuncture'/exp OR 'acupuncture' |
| #2 | 'Acupuncture Therapy':ab,ti or 'Acupuncture, Ear':ab,ti or 'Acupuncture':ab,ti or 'electroacupuncture':ab,ti or 'Pharmacopuncture':ab,ti or 'electroacupuncture':ab,ti or 'Acupuncture Treatment':ab,ti or 'Acupuncture Treatments':ab,ti or 'Treatment, Acupuncture':ab,ti or 'Therapy, Acupuncture':ab,ti or 'Pharmacoacupuncture Treatment':ab,ti or 'Treatment, Pharmacoacupuncture':ab,ti or 'Pharmacoacupuncture Therapy':ab,ti or 'Therapy, Pharmacoacupuncture':ab,ti or 'Acupotomy':ab,ti or 'Acupotomies':ab,ti or 'Acupunctures, Ear':ab,ti or 'Ear Acupunctures':ab,ti or 'Acupuncture, Auricular':ab,ti or 'Acupunctures, Auricular':ab,ti or 'Auricular Acupunctures':ab,ti or 'Auricular Acupuncture':ab,ti or 'Ear Acupuncture':ab,ti or 'acupunc*':ab,ti |
| #3 | #1 OR #2 |
| #4 | 'fatigue'/exp OR fatigue OR 'fatigue':ab,ti OR 'lassitude':ab,ti OR 'tiredness':ab,ti OR 'weary':ab,ti |
| #5 | 'neoplasms'/exp OR neoplasms OR 'neoplasms':ab,ti OR 'tumors':ab,ti OR 'neoplasia':ab,ti OR 'neoplasias':ab,ti OR 'neoplasm':ab,ti OR 'tumor':ab,ti OR 'cancer':ab,ti OR 'cancers':ab,ti OR 'malignant neoplasm':ab,ti OR 'malignancy':ab,ti OR 'malignancies':ab,ti OR 'malignant neoplasms':ab,ti OR 'neoplasm, malignant':ab,ti OR 'neoplasms, malignant':ab,ti OR 'benign neoplasms':ab,ti OR 'neoplasms, benign':ab,ti OR 'neoplasm, benign':ab,ti OR 'benign neoplasm':ab,ti |
| #6 | 'randomized controlled trial':ab,ti OR 'controlled clinical trial':ab,ti OR 'clinical trials as topic':ab,ti OR 'trial':ab,ti OR 'random':ab,ti OR 'placebo':ab,ti OR 'search':ab,ti |
| #7 | #3 AND #4 AND #5 AND #6 |

The Search strategy for the Cochrane Library.

| No | Search history |
| --- | --- |
| #1 | MeSH descriptor: [Fatigue] explode all trees |
| #2 | (Lassitude):ti,ab,kw OR (Tiredness):ti,ab,kw OR (Weary):ti,ab,kw |
| #3 | #1 OR #2 |
| #4 | MeSH descriptor: [Neoplasms] explode all trees |
| #5 | (Tumors):ti,ab,kw OR (Neoplasia):ti,ab,kw OR (Neoplasias):ti,ab,kw OR (Neoplasm):ti,ab,kw OR (Tumor):ti,ab,kw OR (Cancer):ti,ab,kw OR (Cancers):ti,ab,kw OR (Malignant Neoplasm):ti,ab,kw OR (Malignancy):ti,ab,kw OR (Malignancies):ti,ab,kw OR (Malignant Neoplasms):ti,ab,kw OR (Neoplasm, Malignant):ti,ab,kw OR (Neoplasms, Malignant):ti,ab,kw OR (Benign Neoplasms):ti,ab,kw OR (Neoplasms, Benign):ti,ab,kw OR (Neoplasm, Benign):ti,ab,kw OR (Benign Neoplasm):ti,ab,kw |
| #6 | #4 OR #5 |
| #7 | (randomized controlled trial):ti,ab,kw OR (controlled clinical trial):ti,ab,kw OR (clinical trials as topic):ti,ab,kw OR (trial):ti,ab,kw OR (random):ti,ab,kw OR (placebo):ti,ab,kw OR (search):ti,ab,kw |
| #8 | MeSH descriptor: [Acupuncture] explode all trees |
| #9 | (Acupuncture Therapy):ti,ab,kw OR (Acupuncture, Ear):ti,ab,kw OR (Acupuncture):ti,ab,kw OR (electroacupuncture):ti,ab,kw OR (Pharmacopuncture):ti,ab,kw OR (Acupuncture Treatment):ti,ab,kw OR (Acupuncture Treatments):ti,ab,kw OR (Treatment, Acupuncture):ti,ab,kw OR (Therapy, Acupuncture):ti,ab,kw OR (Pharmacoacupuncture Treatment):ti,ab,kw OR (Treatment, Pharmacoacupuncture):ti,ab,kw OR (Pharmacoacupuncture Therapy):ti,ab,kw OR (Therapy, Pharmacoacupuncture):ti,ab,kw OR (Acupotomy):ti,ab,kw OR (Acupotomies):ti,ab,kw OR (Acupunctures, Ear):ti,ab,kw OR (Ear Acupunctures):ti,ab,kw OR (Acupuncture, Auricular):ti,ab,kw OR (Acupunctures, Auricular):ti,ab,kw OR (Auricular Acupunctures):ti,ab,kw OR (Auricular Acupuncture):ti,ab,kw OR (Ear Acupuncture):ti,ab,kw OR (acupunc*):ti,ab,kw |
| #10 | #8 OR #9 |
| #11 | #3 AND #6 AND #7 AND #10 |

The Search strategy for the WOS.

| No | Search history |
| --- | --- |
| #1 | “TS=(Acupuncture OR Acupuncture Therapy OR Acupuncture, Ear OR Electroacupuncture OR Pharmacopuncture OR Acupuncture Treatment OR Acupuncture Treatments OR Treatment, Acupuncture OR Therapy, Acupuncture OR pharmacoacupunture Treatment OR Treatment, pharmacoacupunture OR pharmacoacupunture Therapy OR Therapy, pharmacoacupunture OR autotomy OR autotomies OR acupuncture, Ear OR Ear acupuncture OR Acupuncture, Auricular OR acupuncture, Auricular OR Auricular acupuncture OR Auricular Acupuncture OR Ear Acupuncture)) AND TS=(Fatigue OR Lassitude OR Tiredness OR Weary)” |
| #2 | “TS=(Neoplasms OR Tumors OR Neoplasia OR Neoplasias OR Neoplasm OR Tumor OR Cancer OR Cancers OR Malignant Neoplasm OR Malignancy OR Malignancies OR Malignant Neoplasms OR Neoplasm, Malignant OR Neoplasms, Malignant OR Benign Neoplasms OR Neoplasms, Benign OR Neoplasm, Benign OR Benign Neoplasm)” |
| #3 | “TS=(randomized controlled trial OR controlled clinical trial OR clinical trials as topic OR trial OR random* OR placebo*)” |
| #4 | #1 AND #2 AND #3 |

# Supplementary Figures and Tables

**Figure.S1 Sensitivity analysis plot of PFS**

**
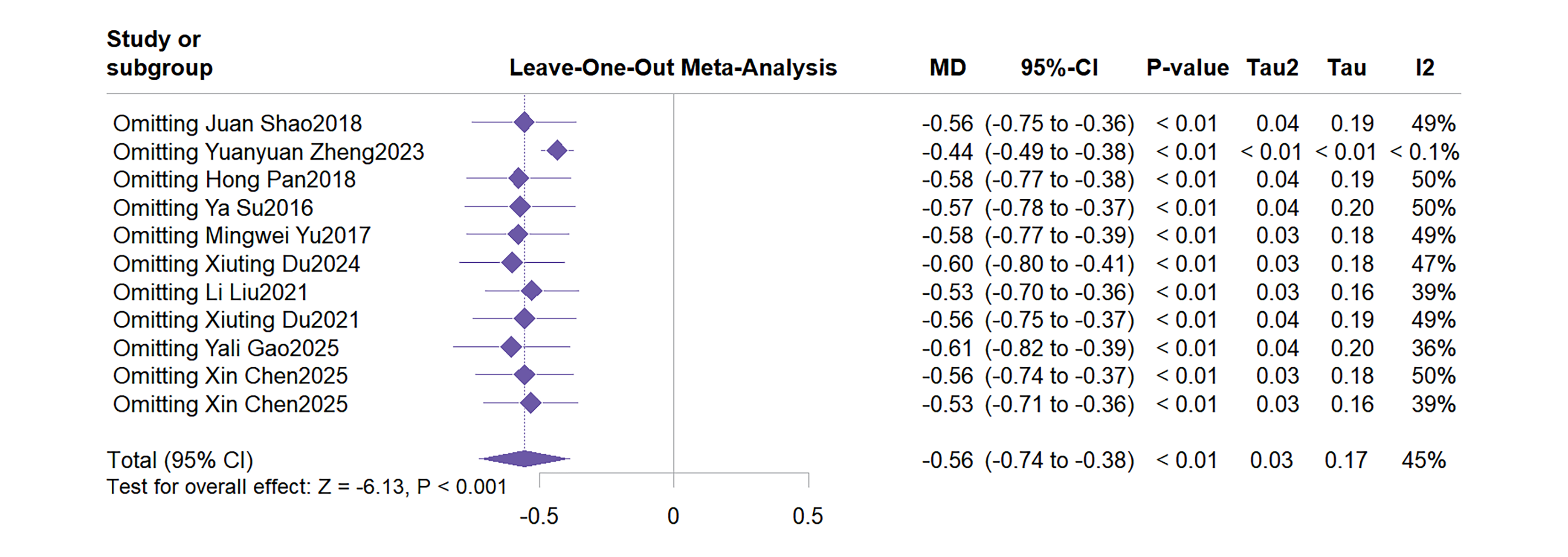
**

**Figure.S2 Sensitivity analysis plot of BFI**


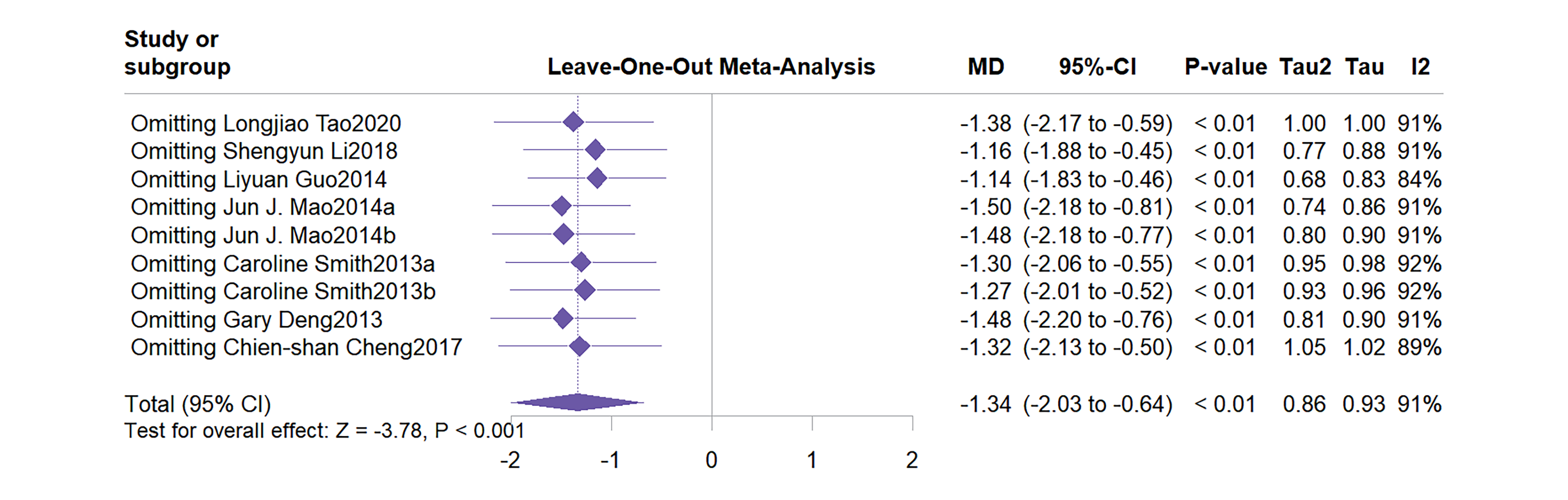


**Figure.S3 Sensitivity analysis plot of CFS**

**
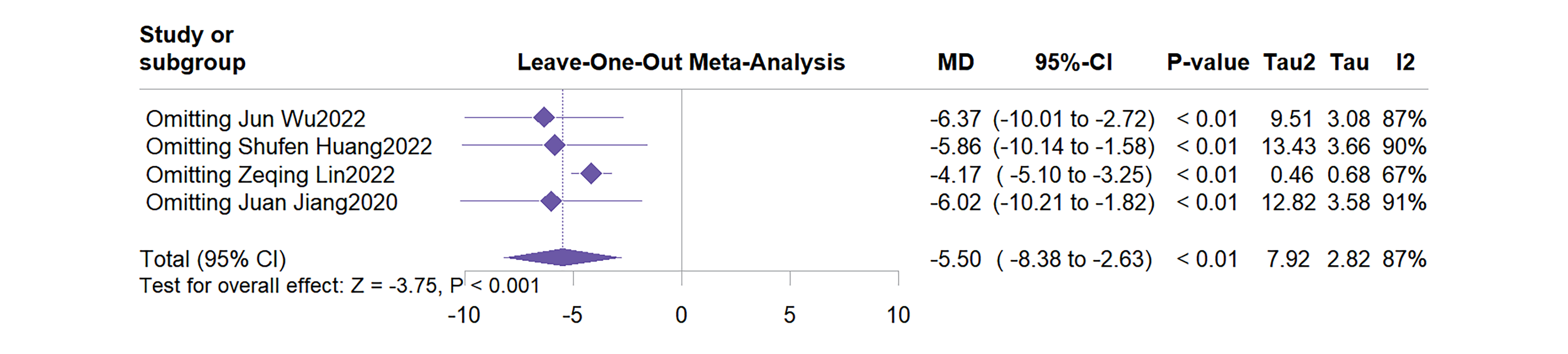
**

**Figure.S4 Sensitivity analysis plot of MFI**

**
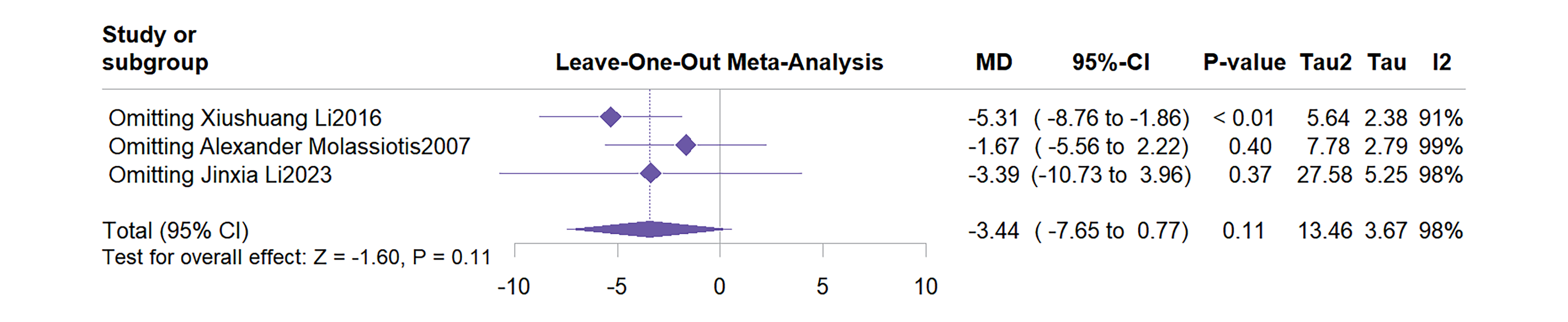
**

**Table.S1 The GRADE System of manual acupuncture compared to usual care for CRF**

**
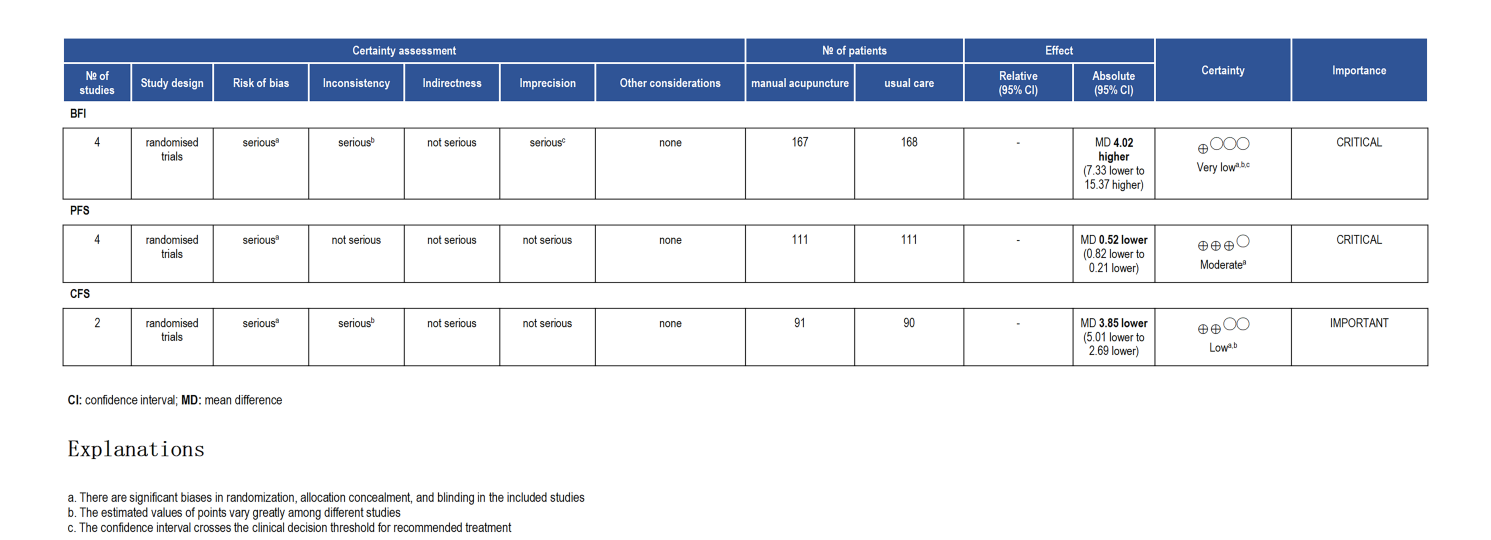
**

**Table.S2 The GRADE System of manual acupuncture compared to Sham acupuncture for CRF**

**
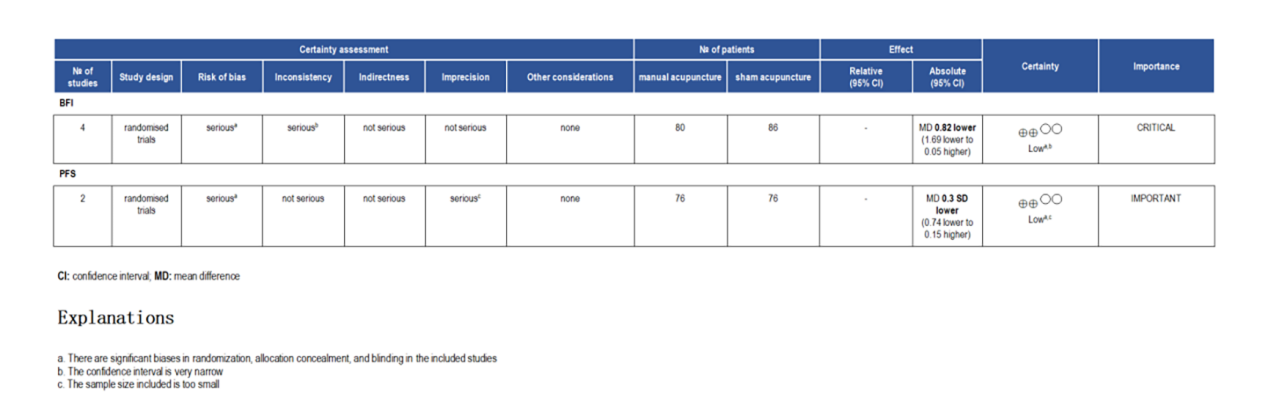
**

**Table.S3 The summary of the sham acupuncture protocols in PFS**

| Author | Acupuncture depth | Acupoint selection | Stimulation method |
| --- | --- | --- | --- |
| Hong Pan | shallow needling | non-acupoint areas | no manual stimulation |
| Mingwei Yu | shallow needling | non-acupoint areas | no manual stimulation |
| Xin Chen | shallow needling | non-acupoint areas | no manual stimulation |

**
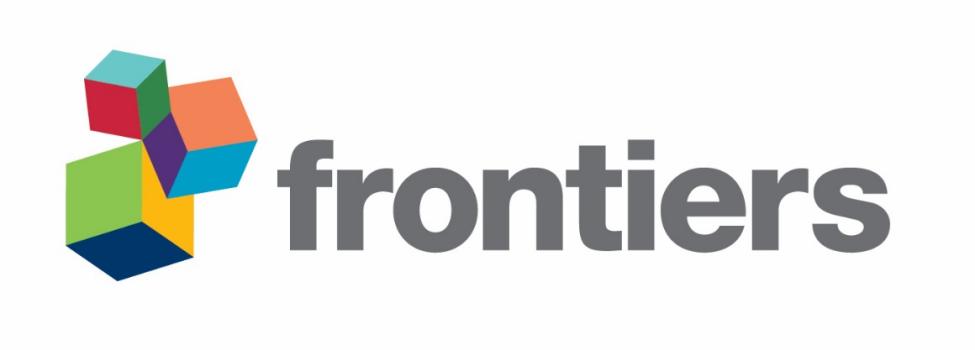
**
